# Co-creating a local environmental epidemiology study: the case of citizen science for investigating air pollution and related health risks in Barcelona, Spain

**DOI:** 10.1186/s12940-021-00826-8

**Published:** 2022-01-12

**Authors:** Florence Gignac, Valeria Righi, Raül Toran, Lucía Paz Errandonea, Rodney Ortiz, Mark Nieuwenhuijsen, Javier Creus, Xavier Basagaña, Mara Balestrini

**Affiliations:** 1 Barcelona Institute for Global Health (ISGlobal), Barcelona, Spain; 2 Universitat Pompeu Fabra (UPF), Barcelona, Spain; 3 CIBER Epidemiología y Salud Pública (CIBERESP), Barcelona, Spain; 4 Ideas For Change (IFC), Barcelona, Spain

**Keywords:** Air pollution, Environmental health, Epidemiology, Civic concerns, Citizen science, Participatory research, Online survey, Study design, Co-creation workshop

## Abstract

**Background:**

While the health risks of air pollution attract considerable attention, both scholarly and within the general population, citizens are rarely involved in environmental health research, beyond participating as data subjects. Co-created citizen science is an approach that fosters collaboration between scientists and lay people to engage the latter in all phases of research. Currently, this approach is rare in environmental epidemiology and when co-creation processes do take place, they are often not documented. This paper describes the first stages of an ongoing co-created citizen science epidemiological project in Barcelona (Spain), that included identifying topics that citizens wish to investigate as regards air pollution and health, formulating their concerns into research questions and co-designing the study protocol. This paper also reflects key trade-offs between scientific rigor and public engagement and provides suggestions to consider when applying citizen science to environmental health studies.

**Methods:**

Experts created an online survey and analyzed responses with descriptive statistics and qualitative coding. A pop-up intervention was held to discuss with citizens their concerns about air pollution and health. Later on, a community meeting was organized to narrow down the research topics and list potential research questions. In an online survey, citizens were asked to vote for the research question they would like to investigate with the experts. A workshop was held to choose a study design in which citizens would like to partake to answer their preferred research question.

**Results:**

According to 488 respondents from the first survey, cognitive and mental health were the main priorities of investigation. Based on the second survey, with 27% of the votes from 556 citizens, the most popular research question was, “How does air pollution together with noise and green/blue spaces affect mental health?”. The study design selected was an observational study in which citizens provide daily repeated measures of different cognitive and mental health outcomes and relate them to the air pollution concentrations.

**Conclusions:**

Based on the co-creation activities and the results obtained, we conclude that applying citizen science in an environmental health project is valuable for researchers despite some challenges such as engaging citizens and maximizing representativity.

**Supplementary Information:**

The online version contains supplementary material available at 10.1186/s12940-021-00826-8.

## Introduction

Poor air quality constitutes a serious health burden worldwide [1]. In the last years, there has been an extensive body of research documenting established and new health effects associated with air pollution [2]. The publication of new scientific evidence about the health risks of air pollution is contributing to raising public concerns. For environmental health experts, the lay people perception of air pollution as an important public health issue and the civic mobilization are two key factors that are able to move from research to action and influence policy changes [3, 4]. Hence, more researchers have started to incorporate participatory practices in order to better align the design of their studies on air pollution and health with public concerns, with the hopes that research results can then lead to actions relevant to the local community needs [5, 6].

Participatory practices can take many forms, from the less participatory practices (in which the lay public solely collects data) to the most participatory (in which the lay public gives their input throughout the entire research process). In the field of environmental epidemiology, research approaches engaging the lay public and affected communities are carried out under several banners such as community-based participatory research, community-driven research, participatory action research, popular epidemiology, and more recently, citizen science [5, 6]. The latter broadly refers to the engagement of the general public (non-professional scientists) in scientific research tasks [7]. In particular, citizen science initiatives adopting co-creation methods (i.e. co-created citizen science) aim to give citizens an opportunity to take part in the decision-making process of all aspects of a research project, such as defining the study questions, developing the data collection tools and analyzing the data [7, 8]. A key contribution to the application of co-created citizen science in research is the Bristol Approach [9, 10]. The Bristol Approach proposes a model of co-creation that builds on the principles of participatory action research, people-centered innovation and the common goods. In a recent narrative review, we adapted this model from a general perspective and developed a four-phase framework with features that occur in different participatory practices in environmental health research: (1) identification (civic concerns are identified and translated into a research question), (2) design (data collection tools, data governance and other aspects of the study protocol are defined), (3) the deployment (data are collected and analyzed) and (4) action (results are transformed into practical citizen-produced knowledge to inform public policies) [11]. This framework closely resembles to other participatory research frameworks and guidelines that have been already defined by environmental health researchers [5, 12, 13]. In comparison to the other frameworks, this four-phase framework stresses the importance of involving the citizens in each phase of the research process, that is the citizens having an active role in the scientific governance, and calls for an equal involvement between experts and citizens when it comes to decision-making [11].

Over the last few years, although there have been several initiatives claiming to apply a citizen science approach to measure air quality parameters [14, 15], participatory research projects on air quality are not new and exist since more than two decades [16–18]. While such initiatives are driven by health-related concerns, those research projects for the study of air pollution using citizen science or other participatory approaches do not often focus specifically on assessing the link between air pollution and health [7, 11]. The research study of Wing et al. [6] is a good example of an environmental epidemiology study in which citizens were involved in almost all the research process and collaborated with researchers to investigate the relationship between exposure to air pollution from industrial swine production and several health endpoints, including lung function, blood pressure, mood and stress level. Community residents were actively involved in identifying research questions, collecting data, recruiting study participants, interpreting and disseminating the results.

Along the lines of participatory research like the work of Wing et al. [6], here, we present the initial phases of the Barcelona CitieS-Health project, an ongoing environmental epidemiology project in which citizens co-design with scientists a study to assess the link between air pollution exposure and health. Following the co-created citizen science framework previously mentioned, the project aims to involve citizens in all phases of the research, including deciding the research question, designing the study, collecting and analyzing data, interpreting and disseminating the results and ultimately, suggesting policy-related actions. As examples of co-created projects are limited, more so in the field of environmental epidemiology, it is important to document all the steps taken as this information can contribute to the design of future projects with higher level of participation in the field. In this paper, we describe the co-creation activities from the first and second phases of the framework, that is the activities that were conducted to collaboratively define the research question and co-design an environmental epidemiological study protocol. Also, we present the main results of each activity and reflect on the added value of civic inputs and the challenges when co-creating an environmental epidemiological study with citizens. Moreover, we provide practical suggestions for environmental health researchers looking to apply co-created citizen science methods to their studies. It is important to note that in this paper, the term “citizen” is used to distinguish the lay public from professional scientific researchers and does not indicate the citizenship status of the people participating in the research project.

## Methods

### Setting

The study presented here is part of CitieS-Health, a project funded by the European Commission under the H2020 program, which aims to implement participatory research methods through the entire scientific process around the topic of urban environmental pollution and health. The project includes five pilot studies in five European cities. Here, we present the pilot study conducted in the city of Barcelona (Spain), which covers the topic of air pollution and health.

Barcelona has pollutant levels above the WHO recommendations [19] and the consequences of air pollution exposure on health are one of local citizens’ preoccupations [20, 21]. As a result, different citizen platforms advocating for cleaner air and projects engaging citizens in collecting air pollution exposure have been developed in the city [22–25].

### Co-designing the research question

The process of co-designing the research question was conducted from August 2019 to January 2020 and included (1) an online survey on knowledge, perceptions and preferences on topics to be investigated around the theme of air pollution and health, (2) a pop-up intervention to approach citizens and discuss their interests and concerns, (3) a community meeting with citizens in order to start formulating potential research questions based on the results of the survey, and (4) a second online survey to identify the most preferred research question to be implemented in the epidemiological study. In Table 1, a list of all activities along with their aims and tools used is provided in a chronological order.

**Table 1:** Co-creation activities conducted in the Barcelona pilot along with their aim and tools used

Phase	Aim	Type of activity	Tool ^**a**^
**Co-designing the research question**	To identify topics citizens are concerned about and would like to investigate in the context of air pollution and health.	Online survey	–
	To raise awareness about the project across all Barcelona districts and collect further qualitative insights on topics citizens are concerned about and would like to investigate in the context of air pollution and health.	Pop-up intervention	In-depth discussion canvas
	To formulate the concerns into potential research questions.	Community meeting	Identification Issues canvas
	To select the research question to investigate.	Online survey	–
**Co-designing the study protocol**	To gauge citizens’ preferences in certain aspects of the study design and to be included in the study protocol.	Workshop	Experiment Design canvas

^a^The templates of the tools can be found in Supplementary Materials. Also, more details on the tools used can be found in the reports available on the website of the CitieS-Health project (https://citieshealth.eu/) and also in the CitieS-Health toolkit (https://citizensciencetoolkit.eu/), which includes ideas and concrete examples on how to engage citizens in citizen science projects in environmental health studies

The first survey was launched alongside a strategic video campaign entitled “Everything you wanted to know about the air but were too afraid to ask”. The invitation proposed the respondents to partake in the design of a scientific study, the first phase of which consisted on collecting citizen concerns and topics of interest regarding research on air pollution and health. To attract a significant number of respondents, a link to the survey was posted through diverse social media platforms (Twitter, Facebook and LinkedIn). Additionally, to ensure widespread dissemination, the link to the survey was sent to key stakeholders including citizen groups concerned about environmental issues, city council representatives, journalists, local governments, and the public transport authority.

The survey was not targeting a specific group of citizens and the questions of the survey were chosen by experts in environmental epidemiology and in civic engagement from the research group. The civic engagement experts were responsible for designing, coordinating and animating activities with the citizens, and had an advanced knowledge in gauging civic concerns, communicating scientific concepts to lay public and stimulating the motivation of citizens to engage in the project. The survey questionnaire was designed for the purpose of collecting (1) citizens’ perception of air quality, (2) concerns over air pollution health impacts and (3) topics of interest to which they would like to conduct scientific research. The complete survey consisted of a total of nine questions and included 5-point Likert scale and free text questions (Supplementary Table 1). Experts designed a short questionnaire in order to maximize participation, but at the same time to collect enough information to identify key topics of concern and interest. A pilot version of the survey was shared among selected contacts and reviewed to make final adjustments before its final implementation.

An offline pop-up intervention across all the districts of Barcelona was also organized during the Parking Day, an annual initiative in which various organizations and communities temporarily transform public parking spaces by giving them a different use, one that promotes a sustainable urban environment [26]. In collaboration with another national citizen science project called Los Vigilantes del Aire [27], strawberry plants were distributed for citizens to measure air pollution at home. Since particulate matters are iron-rich particles and tend to accumulate on the plant leaves, a proxy for estimating the concentration of ambient particulate matter is by measuring the concentration of ferromagnetic particles on the leaves [28]. Plus, this assessment method has been demonstrated to be promising for participatory environmental epidemiology research [29]. This pop-up intervention first aimed to raise awareness about the project across all Barcelona districts, which contributed to overcome the limitations of the distribution of the online survey. It helped to gather information about citizens’ concerns on air pollution and health from different socio-economic areas of the city, and at the same time, invite them to participate in the online survey. More specifically, we had a stand in ten districts of Barcelona and for each stand we used a canvas that we developed for allowing for in-depth discussions. This canvas consisted of a poster with a title “What worries my neighborhood regarding pollution and health?” listing different body parts and health topics. Citizens had to identify the one they would like to know how air pollution affects it using stickers and were asked to explain why. The aim of using this canvas was twofold. It acted as a thematic and social icebreaker. On the one hand, it allowed people to start thinking about the relationship between human health and air pollution, identifying how poor air quality in their neighborhoods was affecting their own body in different ways (causing them to cough, have itchy eyes, be more tired, etc.). On the other hand, because it was a poster, it allowed people to socialize their concerns and find out how others in the same or different neighborhoods felt about air pollution and health. The template of the canvas can be found in Supplementary Material.

Once the results of the survey were analyzed and topics were identified, we organized a community meeting with public authorities, health bodies, experts and citizens to start co-defining a set of possible questions to investigate building on the findings of the survey and the pop-up intervention. During this event, participants first learned about the survey results and then were divided into working groups for further discussions on potential research questions. A tool developed and used during this activity was the Identification Issues canvas (the template of the canvas can be found in Supplementary Material). Each working group was assigned to a thematic table representing one of the most mentioned health topics in the survey. The number of thematic tables depended on the expected number of participants that registered to the event and the results from the first survey and the pop-up intervention. Using the canvas, participants were asked to define a question based on that topic as well as to identify barriers, and opportunities to investigate such question. Moreover, each participant at the table received an “actor card” which is a card representing a population group, such as asthmatics, athletes, elderly people, and children to enrich the perspective of those affected by the health problem at stake. During the community meeting with citizens, the degree of novelty of the different research topics was raised by scientists so that citizens had another piece of information to consider. Moreover, during this meeting, scientists discussed about high-risk hypotheses, i.e., very novel hypotheses that could have a high impact if confirmed but also have a high risk of not being confirmed, which can then reduce the applicability of the results for implementing new policies to reduce air pollution.

Afterwards, scientists and civic engagement specialists collected all the inputs from the community meeting and assessed the viability and feasibility of the different research questions proposed. Since the general goal of the CitieS-Health project was to explore how pollution in the living environment of a group of citizens is affecting their health, questions had to be formulated in a way that the study could assess the relationship of air pollution exposure with a health outcome. The resulting list of questions was shared via a second online survey in which citizens had to vote for their preferred question. The question most voted was selected as the final research question to investigate with scientists.

### Co-designing the study protocol

The process of co-designing the study protocol was conducted from February to April 2020. Based on the research question most voted by the citizens in the second online survey, a workshop was organized to determine with citizens different elements of the study design and data collection protocol of the research project. A tool that was developed for this event was the Experiment Design canvas (the template of the canvas can be found in Supplementary Material). This tool allowed epidemiological researchers to present and discuss with citizens different types of epidemiological studies and gauge their preferences about different aspects of study design. Specifically, the canvas is composed of different posters, each representing a type of epidemiological study. Experts presented to citizens three types of epidemiological study (observational/panel, experimental and cross-sectional) while explaining their strengths and limitations. The description of each study was explained using simple terms and without belaboring all the aspects to consider when designing a research study. The panel study was described as a study that was requiring participants to collect data (e.g. answering a self-report questionnaire) one or more times per day for one week or more. It was explained that each participant was serving as his or her own control and the aim was to compare the health outcomes between the days more polluted and the days less polluted. It was highlighted that this type of study was effective for studying short-term health effects of air pollutants but could be time-consuming if a daily questionnaire had to be completed. The experimental study was described as a study in which participants had to alter their daily routine to follow specific instructions, for example a study in which participants are invited to walk for two hours and the same distance in a street known to be more polluted (e.g. busiest shopping street) and, in another day, in a park or area in Barcelona known to be less polluted (thus modifying their routines). On the one hand, it was explained that this experimental design was requiring a bit more involvement from the participants and in the given example, the experimental conditions (busy street vs. park) could not be blind, and consequently, impossible to avoid a placebo effect, an important bias in research. On the other hand, it was suggested that this study could produce results less prone to alternative explanations and could generate a higher exposure contrasts and thus, make it more likely to detect effects. For the cross-sectional analysis, experts proposed to the citizens to use a postal or online questionnaire that could be sent to Barcelona residents to ask about health conditions (e.g., if they have certain disease/symptoms or not) and to assess exposure to air pollution using the home address of participants. This was presented as a quick, cheap and easy to conduct study in which multiple outcomes could be measured. Plus, it was presented as a study design that was able to detect some associations, but for which, in comparison to the other designs, is usually more difficult to see if the associations are causal, or to identify which is the cause and the effect between the exposure and the outcome. The three posters for each type of study were displayed on a wall and citizens were first invited to choose individually the type of study they were interested in to partake based on the time and effort they were willing to dedicate. Each poster had three different rows, corresponding to different aspects to be discussed step-by-step with participants. In the first row, participants were invited to select the aspect of the health topic they were most interested in. In the second row, participants had to reflect on the kind of data they were willing to collect and in the third row, they had to choose or propose tools for collecting the data. To vote, citizens used stickers. While the participants were voting, experts initiated more individual conversations with the participants to further discuss their preferences in other elements of the study design, including the participation duration, indicators of the most popular health topics and the tools to collect health and environmental exposure data. Citizens were invited to write their preferences on the posters. The final study protocol was written by the scientists taking into account all citizens’ inputs, made available online and shared with a selected group of citizens for feedback.

### Data analysis

For both the first and second online survey, network ID and email addresses were verified to control for duplication. Respondents’ postal codes were matched to the corresponding district. Only Barcelona residents were included in the analysis. Number of respondents and frequencies were reported to describe the results. All analyses were conducted with the STATA 16.0 statistical software package (College Station, TX). The dataset containing anonymized results from questions 1 to 6 of the first survey is available for download and free use through the file repository Zenodo [30].

In the first survey, perception rates were averaged at the district level and plotted on a map, which was compared to a map of NO_2_ levels. Answers from the two open-ended questions were analyzed using a content analysis approach [31]. Specifically, word frequency analysis was used to explore responses from question 7, that is summing the number of times particular words appear in the respondents’ answers [32]. Key words identified were those regarding specific health issues or human body parts and functions as well as verbs qualifying an effect. Words of the same derivation or meaning (e.g. cardiac/cardiovascular; odor/olfaction, fertility/infertility) were linked. However, related words (e.g. lungs/respiratory) were not linked in order to avoid overgeneralization of the vocabulary chosen by the respondents.

To analyze open-ended responses from question 8, we used an open coding process to capture the most recurrent ideas from the respondents. Answers were labeled by a code summarized by one or two words representing the main aspect. Multiple codes were applied when respondents’ answers addressed more than one different topic [33]. Codes were then grouped together into categories. Only one category (“Health effects”) was deductively defined and the rest of the categories were created inductively. Results were summarized by reporting category frequencies.

## Results

For the first survey, a total of 488 out of the 582 respondents were living in the ten districts of Barcelona City. For three respondents from Barcelona it was not possible to know in which district they were living in. Respondents with missing data were not excluded. The majority of the respondents were living in Eixample (31.5%), Gràcia (14.6%) and Sant Martí (14.2%) districts (Table 2). Respondents were mainly young and middle-aged adults, where approximately 64% were aged between 28 and 47.

**Table 2:** Socio-demographic characteristics of the respondents from first and second online surveys

Characteristics	Survey I (***n*** = 488)^**a**^		Survey II (***n*** = 560)	
	n	%	n	%
**Age groups**				
Less than 18	1	0.36	11	1.98
18-27	24	8.63	82	14.75
28-37	83	29.86	163	29.32
38-47	96	34.53	157	28.24
48-64	60	21.58	126	22.66
More than 64	14	5.05	17	3.06
*Total*	*278*	*100*	*556*	*100*
**District**				
Eixample	153	31.35	148	26.62
Gràcia	71	14.55	95	17.09
Sant Martí	69	14.14	68	12.23
Sants-Montjuïc	52	10.66	59	10.61
Ciutat Vella	36	7.38	39	7.01
Horta-Guinardo	35	7.17	39	7.01
Sarrià Sant-Gervasi	28	5.74	51	9.17
Sant Andreu	17	3.48	23	4.14
Nou Barris	15	3.07	22	3.96
Les Corts	9	1.84	10	1.80
District N/A	3	0.61	2	0.36
*Total*	*488*	*100*	*556*	*100*

^a^A total of 210 (43%) respondents did not answer the question

Almost 65% of the respondents perceived the air as considerably polluted (37.50%) or highly polluted (27.46%) (Question 3). This proportion went up to 95% when including those who perceived the air as moderately polluted (29.30%). Citizens’ perception of air quality per district followed a similar pattern than the actual NO_2_ concentration levels in Barcelona (Fig. S1).

Table 3 presents the summary statistics for questions 4, 5 and 6. The situations where respondents felt that air pollution affected them the most were when they walk in the street (75.8%), and when they go by bicycle (43.7%) (Question 4). Well over 69% of the respondents were interested to know more about how air pollution can affect the respiratory system (Table 3, question 6). Other health topics of interest selected were concentration and development (40%), heart and arteries (37%) and stress (33%). The answers given in the option ‘Other’ are described in Supplementary Table 2.

**Table 3:** Descriptive summary of the perceived situations where pollution affects the most (Q4), perceived vulnerable population groups to air pollution (Q5) and health topic of interest for more investigation (Q6) (N respondents = 488)

Questions^**a**^	Answers	n	% of responses^**b**^	% of cases^**c**^
Q4: In which situations do you perceive that air pollution affects you the most?	While walking in the street	370	32.51	75.82
	While going by bicycle	213	18.72	43.65
	While walking with children	150	13.18	30.74
	While doing outdoor sports	144	12.65	29.51
	While traveling by metro	106	9.31	21.72
	When I am at home	79	6.94	16.19
	When I drive	48	4.22	9.84
	When I am at work	15	1.32	3.07
	Other	13	1.14	2.66
	*Total*	*1138*	*100*	*233.20*
Q5: Are you worried about how air pollution affects any of these groups?	Children	388	28.87	79.51
	Elderly people	225	16.74	46.11
	People with asthma or respiratory problems	220	16.37	45.08
	Pregnant women	186	13.84	38.11
	Pedestrians	131	9.75	26.84
	People with allergies	91	6.77	18.65
	Deliverymen using motorbike	41	3.05	8.40
	Sportsmen/women	29	2.16	5.94
	Other	19	1.41	3.89
	Students	14	1.04	2.87
	*Total*	*1344*	*100*	*275.41*
Q6: You would like to know how air pollution affects …	Respiratory system	337	24.16	69.06
	Concentration and development	191	13.69	39.14
	Heart and arteries	173	12.40	35.45
	Stress	167	11.97	34.22
	Mental health	123	8.82	25.20
	Ageing	121	8.67	24.80
	Fertility / Reproductive system	75	5.38	15.37
	Allergies	70	5.02	14.34
	Digestive system	52	3.73	10.66
	Hair / Skin	42	3.01	8.61
	Sport performance	33	2.37	6.76
	Other	11	0.79	2.25
	*Total*	*1395*	*100*	*285.86*

^a^Q4, Q5 and Q6 have no missing cases

^b^The “% of responses” column reports percentages with respect to the overall sum of responses. For example, 32.51% of all responses of Q4 are “When walking in the street”

^c^The “% of cases” column reflects the average number of responses per respondent (multiplied by 100). For example, 69% of the respondents selected “Respiratory system”

From the answers of a total of 466 respondents describing the effects air pollution on their health (Question 7), a total of 192 key words were counted, in which 32 high frequency words (i.e. repeated 10 times or more) were retrieved and listed in Supplementary Table 3. The most frequent health-related word reported referred to the respiratory system (312 times). Other words often repeated were related to cardiovascular health (80 times) and to stress (75 times). Additionally, if the topic of development was combined with the one of cognition into one single keyword, it represented a significant health concern of citizens (84 times).

Based on 452 answers about what citizens would like to investigate on air pollution and health (Question 8), a total of 92 codes were created and regrouped into five categories. Codes and categories are summarized by frequency count in Table 4. The first category, ‘Health-related outcomes’ addresses the array of health elements reported by the respondents. A recurrent code was the one of ‘Overall health’, which corresponds to when respondents tended to express health as a whole or were interested in all health topics. Also, respondents often used the term “mental health” by echoing other concepts such as mood, anxiety and development: “*How much it affects our moods or mental states*” / “*The impact on mental health and cognitive skills and concentration*”. One respondent mentioned the concept of mental health but referring to cognitive decline and dementia: “*The mental health loss around the age of 60*”, whereas another expressed mental health more as an emotional state: “*How pollution affects people’s mental state (safety, self-esteem, anxiety)*”.

**Table 4:** Codes and categories deriving from responses to question 8 of the first online survey

	Codes (n)^**b**^		
**Health-related outcomes (44)**			
Overall health	(39)	Degenerative diseases	(2)
Respiratory system	(30)	Emotions	(2)
Cancer	(16)	Food/Nutrition	(2)
Life expectancy	(12)	Happiness	(2)
Mental health	(12)	Tumor	(2)
Stress	(11)	Anxiety	(1)
Development	(9)	Blood	(1)
Allergies	(8)	Bones	(1)
Brain	(7)	Chronic disease	(1)
Fertility/Reproductive	(7)	Digestive system	(1)
Mortality	(7)	Endocrine system	(1)
Quality of life	(7)	Genetic	(1)
Skin	(7)	Hair	(1)
Ageing	(6)	Headache	(1)
Cardiac system	(6)	Hormonal system	(1)
Mood	(4)	Inflammation	(1)
Neuro	(4)	Irritability	(1)
Concentration	(3)	Metabolism	(1)
Immune system	(3)	School performance	(1)
New health topics	(3)	Tiredness	(1)
Asthma	(2)	Urine	(1)
Cognition	(2)	Well being	(1)
**Exposure assessment (8)**			
Mitigation measures	(85)	Schools	(5)
Sources	(34)	Atmospheric conditions	(1)
Pollutants	(15)	House	(1)
Urban features	(6)	Noise	(6)
**Target population groups (13)**			
Children	(46)	Asthmatic and allergic people	(1)
All groups	(11)	Pedestrians	(1)
Pregnant women	(9)	People with respiratory problems	(1)
Elderly people	(5)	Sportsmen	(1)
Animals	(2)	Students	(1)
Cyclists	(2)	Women	(1)
Healthy people	(2)		
**Study design (14)**			
Comparison	(32)	Impact evaluation	(2)
Long term	(14)	Individual level	(2)
Direct (unmediated)	(9)	City level	(1)
Cohort studies	(4)	Independent	(1)
Short term	(3)	Local level	(1)
Time (day) of exposure	(4)	Interdisciplinarity	(1)
Expert opinion	(2)	National scale	(1)
**Advocacy (3)**			
Political changes	(9)	Bottom-up initiatives	(1)
Visibility	(5)		

^a^Total number of codes

^b^Total number of times code is repeated. Question what about what citizens would like to investigate about air pollution and health. The coding was based on a total of 452 responses

The second category, ‘Exposure assessment’, accounted for when respondents discussed about evaluating measures to reduce air pollution, identifying specific air pollutants (e.g. NO_2_, CO_2_, O_3_) or specific air pollution sources (e.g. cars, motorbikes, boats) and were considering other factors that could influence health such as noise. A shared opinion amongst 19% of the citizens (85 ‘Mitigation measures’ codes out of 452 responses) was that research should prioritize studying effective mitigation measures that could reduce air pollution and thus, minimize adverse health effects.

The third category, ‘Target population groups’ highlights specific groups of people whom citizens would like to focus the research on. Children appeared in more than 46 responses of the respondents and these groups were often related to developmental health and school environment.

The fourth category entitled ‘Study design’, refers to respondents’ discussing methodological aspects of an epidemiological study. The two main aspects were about adopting a comparative approach (e.g. urban vs rural, healthy vs unhealthy people) and observing effects over a long period of time. Another element touched upon a scientific concept called the control of confounding, which in the responses of the citizens can be translated by this desire to find “*direct*”, “*clear*” and “*unmediated*” relation between the air pollution exposure and the health outcome.

Finally, the fifth category, ‘Advocacy’, underscores a shared desire amongst citizens to use the results of the investigation to beget political change, raise awareness and support bottom-up initiatives. As one respondent stated: “*Whatever is necessary for changes to occur.*” Also, one respondent suggested investigating a new topic that substantiates the urgency to take actions: “*Something new and that forces urgent action on politics*.”

Regarding the pop-up intervention, around 1200 citizens were reached out. The concerns and interests expressed by them were similar to the main results found in the first online survey (data not shown). Moreover, a total of 40 citizens attended the community meeting. Based on the results of the first survey and the pop-up intervention, four thematic tables were prepared: two on air pollution and mental health and two on air pollution and respiratory system. In particular for the topic of mental health, since citizens were reporting terms such as concentration, stress, anxiety, cognition sometimes like synonyms in the first survey, it was more practical for scientists to present all these domains under the umbrella of mental health. For both tables on air pollution and mental health, citizens proposed a similar question that aimed to investigate how stress, happiness and other aspects of mental well-being could be affected by not only air pollution but other environmental exposure such as noise and greenspaces. An opportunity they saw in investigating such question was that its results could serve for the Barcelona City Council, which currently has a clear political priority to improve psychological well-being and understand the determinants of mental health in the city. The main barriers of this research question identified by the citizens were more on a methodological level, for example, the risk of subjectivity if mental health was self-reported and the risk of excluding some communities lacking technological skills or access to technology (e.g. elderly people or people with basic cellphones) if mental health had to be measured using mobile application. In the two tables on air pollution and respiratory health, citizens were interested in investigating if there was a relationship between exposure to air pollution and respiratory health based on the time spent on the street and if this was also affected by the presence of natural spaces. One opportunity highlighted was the possibility to identify streets more contaminated and thus streets to avoid walking. Citizens also proposed to investigate if for people doing outdoor sports, their performance (using respiratory parameters) was affected. Another suggestion was to investigate the effect of air pollution on cardiorespiratory health and explore if this effect was impacted by wearing or not wearing a mask. Barriers identified by the citizens in both tables were also mainly methodological, for instance, they thought that measuring respiratory health is more complex and engaging a lot of participants willing to share their routes or having to wear a mask may be more difficult.

All in all, this meeting helped scientists to formulate a list of eight possible questions to investigate (Table 5). The questions addressed recurrent topics covered during the pop-up intervention and community meeting, including cardiorespiratory and, cognitive and mental health (stress, mood and concentration), physical activity as well as other factors (noise, green and blue spaces) that could impact health. This list of eight questions was included in the second online survey. A total of 556 participants living in Barcelona responded and shared similar characteristics as those who responded to the first survey (Table 2). The most voted question was: “How does air pollution together with noise and green/blue spaces affect mental health?”

**Table 5:** List of potential research questions citizens would like to investigate based on the community meeting

Potential research questions	*n* (%)
How does air pollution together with noise and green/blue spaces affect mental health?	153 (27.52)
How does air pollution together with noise and green/blue spaces affect cardiorespiratory health?	132 (23.74)
Does air pollution affect levels of stress and/or emotional state?	115 (20.68)
How does wearing a mask or without a mask affect my cardiorespiratory health?	45 (8.09)
Does air pollution affect my sense of well-being when doing physical activity?	42 (7.55)
Does air pollution affect my cardiorespiratory health when doing physical activity?	36 (6.47)
Does air pollution affect concentration and/or productivity?	21 (3.78)
Does air pollution affect my performance when doing physical activity?	12 (2.16)

There were 50 citizens participating in the co-creation workshop on study design. Based on discussion and the results from the posters, the study design most preferred by the citizens was an observational panel study. Scientists proposed that participants could provide daily repeated measures of different mental and cognitive outcomes and relate them to the air pollution concentrations. Participants selected the study type based on the effort they were willing to dedicate and the expected results they wish to receive. Regardless of the type of study, self-perceived stress and capacity of attention were among the most preferred and discussed cognitive and mental health outcomes to investigate (Supplementary Fig. 2). Furthermore, citizens expressed an interest in using a mobile application to collect mental health data through games and questionnaire. A number of participants also said they wished to receive more personalized results and to be able to monitor their personal exposure to air pollution. After publishing the protocol online, citizens did not give more feedback. All the above-mentioned preferences were incorporated in the official study protocol and were implemented.

## Discussion

This paper described the first stages of an environmental epidemiology research (Cities-Health Barcelona) using a co-created citizen science approach in the design of the research question and study protocol. Based on the different co-creation activities, including two online surveys, a pop-up intervention, a community meeting and a workshop, we reported what citizens would prioritize investigating in the context of air pollution and health and how they would like to design the research. The development of the co-creation activities and the results obtained led us to identify some added values and the challenges of co-creating research with citizens. In this section, we discuss our findings articulated in terms of two general trade-offs we experienced in applying citizen science in an environmental health project. The first one refers to the balance between the role of scientists as facilitators, the civic inputs and citizens’ control over decisions. The second trade-off touches upon the need to engage citizens versus the need for maximizing quality and representativity in research. To alleviate as much as possible such tensions, we proposed some suggestions that are important for the environmental health research community to consider.

In CitieS-Health Barcelona, co-defining the research question involved combining the input of both citizens and professional scientists. However, citizens and scientists did not have the same control over all of the collective decision-making processes. In our project, we started with an online survey in which citizens had no input from scientists (other than having designed the survey), which allowed us to gauge what was genuinely concerning the citizens and which topics they would like to investigate according to their preoccupations and interests. By doing so, we uncovered interesting findings. When asked about which health topic they were concerned and would like to know more about (Question 6 and 7 from the first online survey), citizens chose mostly respiratory health. Noting the fact that citizens wish to know more about the effects of air pollution on respiratory health suggests that scientific studies’ findings do not reach the public in a meaningful way. In fact, from an experts’ perspective, respiratory health is one of the most covered outcomes in the epidemiological literature and probably one of the first to be linked with atmospheric air pollution. Likewise, a high percentage of citizens wished to know more about the effects on cardiovascular health, when these effects are well established in the scientific community and considered to be the ones causing the biggest burden [34]. Citizens also reported being interested in investigating concentration and development, stress and mental health in general. Despite recent findings, cognitive and mental health remains a novel topic in air pollution research where many gaps still need to be explored [2].

Although the exchange between expert’s knowledge and civic inputs is important, it can be sometimes challenging and the equal involvement between experts and citizens that is promoted by the four-phase framework was difficult to attain. For the first survey, it was important to not overly restrain the citizens to a limited choice of answers, even if scientists knew beforehand that some topics would be more difficult to investigate due to the resource availability and the time constraint of the project. However, at the stage of articulating the questions to investigate during the community meeting, there was a need to balance the viability of the research questions and citizens’ interests. Here, the role of the scientists as facilitators was critical for raising awareness among citizens about what were the implications if a topic was to be investigated and what was the project capacity to support such research project. For instance, an important number of citizens considered that the population at most risk were children (results from the first survey and pop-up intervention). Though this was a clear matter of concerns amongst Barcelona residents, experts decided to not restrict the list of research questions to a specific population. Having minors on board as research subjects and as co-investigators was thought to be more ethically and logistically challenging. In this case, scientific experts were maintaining control over deciding which were the potential questions to be voted. Another example in which the scientists had an important role was when describing the study design. Each type of study was briefly described to avoid boring the public with too much technical details and at the same time, to leave a bit of space for creativity. Based on our experience with the community meeting, we encourage researchers who want to apply co-creation methods to not avoid giving their input by fear of perpetuating a top-down approach to research, since their role as facilitators guide the development of a high quality study. Nevertheless, if decisions have to be made contrary to what is suggested by the citizens, researchers should justify and communicate the inconsistencies in a fully transparent manner.

Similarly, writing the research protocol needed to be elaborated and finalized by scientists after the inputs received from the co-creation workshop. As the actual writing of a study protocol is normally a laborious step requiring precision, it was thought to be too demanding for citizens to be involved in such a task. In fact, one reason we believe we did not receive additional feedback from citizens after publishing the protocol online was of the density and technicality of the document. We found that choosing key elements of the protocol with citizens was enough to align with their needs and interests. To discuss this begs the question of how civic decision-making should proceed in each step of an epidemiological study applying co-created citizen science. Nevertheless, we believe that applying several participatory activities requiring citizens inputs allowed citizens to exert a certain control over the general direction of the research. Other practices could be implemented, such as involving one or a few representatives of citizens in a more laborious task like writing the study protocol, or organizing a committee of citizens that reviews and approves draft and final versions of the protocol [35, 36]. Based on our experience, we would suggest developing several co-creation workshops for the design of the study, rather than just one as we did. For instance, our workshop was centered more on outcome assessment than on exposure assessment, thus a second workshop on how to measure exposure to environmental factors could be relevant. Plus, a group of citizens could review and follow up all the versions of the study protocol until the final one. Scientists would remain responsible for writing the study protocol but could gather more inputs from citizens. This would ensure a better alignment of the study protocol with citizens’ priorities and needs.

A second trade-off we experienced was between the desire of having a representative sample and reaching a maximum of citizens in the project. Representativeness is an issue that can be discussed at different levels in a citizen science project in environmental epidemiology. First, on the epidemiological part, the need for representativeness has been a subject of debate among experts, and one of the main conclusions is that the appropriate selection of participants depends on the objective of the study [37]. Second, one of the rationales of citizen science is that it allows citizens to shape research agendas by choosing or having a say in deciding research topics, as done in the present study. In having a “democratization of science” view, representativeness, or at least a broad inclusion of different population groups, is also a desirable aim, and one of the current challenges citizen science projects are facing [8]. In this sense, our project likely failed to include some population groups, and this should be viewed as a limitation. In fact, the first survey was distributed to stakeholders, some in forms of associations, already involved in air pollution public campaigns and composed of people following the social media of the health research institute in charge of this survey. Therefore, it is probable that the survey principally attracted people who are already aware of air pollution health risks and who have a desire to expose their concerns. However, regardless of diversity in participation, having a high number of contributors has several advantages [8], and our study aimed at reaching the maximum number of participants by designing short surveys. Again, our survey did not allow us to collect in depth sociodemographic data, another limitation of our study. Third, citizen science projects can be considered as a tool for mobilized citizen groups to base their needs for locally generated scientific findings. This situation probably reflects our sample, as participants likely included citizens concerned about poor air quality in the city. Still, we believe it is of interest to report what mobilized groups of citizens believe are the relevant research questions to be investigated, and what is their perception about the topic. It is often the case that small groups of citizens lead the way for societal changes and therefore it remains noteworthy to know their insights.

Furthermore, at the onset of starting the co-identification phase, no actual participant recruitment was required. This led the project to have an informal follow-up of the citizens involved in the different co-creation activities (i.e. online surveys, pop-up intervention, community meeting and workshop). That also means that citizens started to be involved at different stages of the project. Thus, each activity had its own population sample. These samples had also probably different socio-demographic characteristics considering that the time and setting varied from one activity to another. For instance, the workshop was conducted in the evening at the center of the city, which is less practical for adults with young children, whereas the pop-up intervention was held in all the districts during the whole day. Though this could be seen as an issue, it rather reflects the flexibility and openness citizen science brings to research. The co-identification and co-designing phases are meant to grasp the heterogeneity of concerns among the community and thus, creating an exclusive group of citizens at the start of the project could limit gathering relevant unique civic insights. Nevertheless, our suggestion for researchers would be to keep track of who is attending the events and to do this at the very beginning of the project. This would help researchers to have a better idea of the population sample profile for each activity, and in return be able later to better contextualize the results obtained.

Moreover, conducting a rigorous quantitative or qualitative analysis of the civic inputs from the co-creation activities can be challenging for environmental health experts. During the co-creation workshop on study design in which citizens did not necessarily follow every steps of the activity, and thus lead to several inconsistencies in the numbers of votes in the Experiment Design canvas. This happens frequently during co-creation activities and consequently, environmental health researchers should get trained if they want to get the most out of these activities or, they could partner with experts in civic engagement that can gauge better the results from those co-creation activities [11].

The results from the first stages of the Cities-Health project in Barcelona are not without limitations. The lack of diversity amongst respondents in terms of districts and age groups reduces representativeness. Consequently, it is possible that these concerns do not correspond with the concerns of groups from different socio-demographic classes in the city. Moreover, data regarding science literacy, ethnicity and other socio-economic characteristics such as education level, income and job position were not collected, which did not allow us to properly characterize our sample from both online surveys. We did not collect these data to minimize the information collected to facilitate participation. In fact, having to fill out a long survey may annoy some curious citizens who just want to get involved quickly. In addition, during the face-to-face meetings we wanted to build an informal environment to promote an equal relationship between scientists and citizens. In this sense, it was considered odd to ask socio-economic characteristics to the attendants of a workshop. However, this trade-off should be critically analyzed, as scientific publications may require such characterization of the sample, and evaluations of citizen science projects consider diversity as an important indicator.

Throughout this discussion, we highlighted some of the added value and challenges in applying co-created citizen science in environmental epidemiology research project. More details on the specific activities and tools described in this paper can be found in the reports available on the website of the CitieS-Health project (https://citieshealth.eu/). Also, we developed the CitieS-Health toolkit (https://citizensciencetoolkit.eu/), in which we described every steps of the co-creation activities and included some concrete examples on how to engage citizens in citizen science projects in environmental health studies.

## Conclusions

This paper describes the co-creation process of an environmental epidemiology study in the context of urban air pollution and health, which aimed to involve citizens in deciding what to study and how to do it. In the CitieS-Health Barcelona pilot, we found that civic concerns were mainly about the effects of air pollution on different aspects of mental health. Citizens voted for a research question that also aimed to assess other effects of environmental exposures that include noise and green/blue spaces. Despite the fact that the representativeness of these results is subject to limitations, the co-creation activities enabled citizens to provide extensive inputs to the project and are still valuable to inform researchers on current community concerns. The co-designing processes described can be useful for other environmental health researchers who want to apply a co-created citizen science approach in their studies. Moreover, in the context of environmental health research where there are not many documented examples to learn from, we believe that the trade-offs identified between scientific rigor and public engagement and the suggestions provided are important for the research community to consider. However, future work should be done to better understand the optimal application of co-created citizen science in research studies in the field of environmental epidemiology.

## Supplementary Information

**Additional file 1: Tool template 1.** In-depth discussion canvas. **Tool template 2.** Identification Issues canvas. **Tool template 3.** Experiment Design canvas. **Table S1.** List of questions from the Barcelona CitieS-Health Pilot Survey 2019. **Table S2.** Translated answers from option ‘Other’ in Q4, Q5 and Q6 for respondents living in the 10 districts of Barcelona. **Table S3.** List of high frequency health-related and effect-related words (≥ 10 times) from Q7 among respondents living in Barcelona sorted by frequency. **Figure S1.** (a) Subjective perception of air quality (higher rate indicates a perception of a higher level of air pollution) by district in Barcelona; (b) Modeled NO_2_ concentrations in Barcelona in 2017, aggregated at district level. **Figure S2.** Results of the co-creation workshop for each type of study design: (a) Panel / Observational, (b) Experimental and (c) Cross-sectional.

## Data Availability

The dataset (dataset containing anonymized results from questions 1 to 6 of the first survey) generated and analysed during the current study is available in the Zenodo repository [10.5281/zenodo.3886598]. All other data generated and analysed (from the second online survey and workshop) during this study are included in this published article and its supplementary information files.
