# Supplementary material for: Co-creating a local environmental epidemiology study: the case of citizen science for investigating air pollution and related health risks in Barcelona, Spain
Source: Environ Health. 2022 Jan 12;21:11. doi: 10.1186/s12940-021-00826-8 (PMC8753829; doi:10.1186/s12940-021-00826-8)
Supplement: Supplementary file 1 — Additional file 1: Tool template 1. In-depth discussion canvas. Tool template 2. Identification Issues canvas. Tool template 3. Experiment Design canvas. Table S1. List of questions from the Barcelona CitieS-Health Pilot Survey 2019. Table S2. Translated answers from option ‘Other’ in Q4, Q5 and Q6 for respondents living in the 10 districts of Barcelona. Table S3. List of high frequency health-related and effect-related words (≥ 10 times) from Q7 among respondents living in Barcelona sorted by frequency. Figure S1. (a) Subjective perception of air quality (higher rate indicates a perception of a higher level of air pollution) by district in Barcelona; (b) Modeled NO2 concentrations in Barcelona in 2017, aggregated at district level. Figure S2. Results of the co-creation workshop for each type of study design: (a) Panel / Observational, (b) Experimental and (c) Cross-sectional. [file 12940_2021_826_MOESM1_ESM.docx]

**SUPPLEMENTARY MATERIAL**

Co-creating a local environmental epidemiology study: the case of Citizen Science for investigating air pollution and related health risks in Barcelona, Spain.

***Authors:*** *Florence Gignac,Valeria Righi, Raül Toran, Lucía Paz Errandonea, Rodney Ortiz, Mark Nieuwenhuijsen, Javier Creus, Xavier Basagaña and Mara Balestrini*

**Tool template 1:** In-depth discussion canvas

**Tool template 2:** Identification Issues canvas

**Tool template 3:** Experiment Design canvas

**Table S1.** List of questions from the Barcelona CitieS-Health Pilot Survey 2019.

**Table S2.** Translated answers from option ‘Other’ in Q4, Q5 and Q6 for respondents living in the 10 districts of Barcelona.

**Table S3.** List of high frequency health-related and effect-related words (≥ 10 times) from Q7 among respondents living in Barcelona sorted by frequency.

**Figure S1.** (a) Subjective perception of air quality (higher rate indicates a perception of a higher level of air pollution) by district in Barcelona; (b) Modeled NO_2_ concentrations in Barcelona in 2017, aggregated at district level.

**Figure S2.** Results of the co-creation workshop for each type of study design: (a) Panel / Observational, (b) Experimental and (c) Cross-sectional.

**Tool template 1: In-depth discussion canvas**


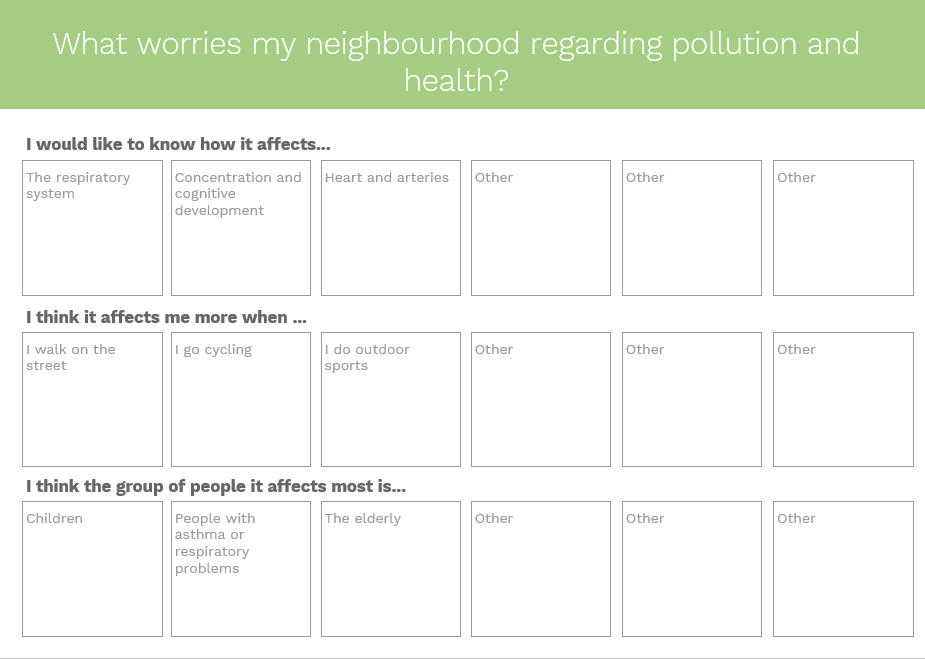


**Tool template 2: Identification Issues canvas**


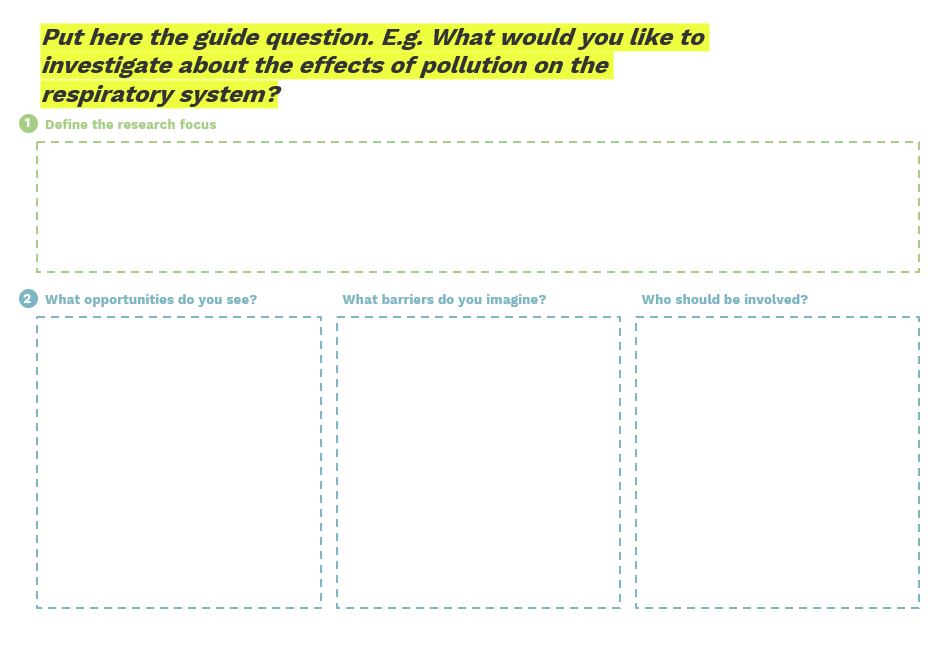


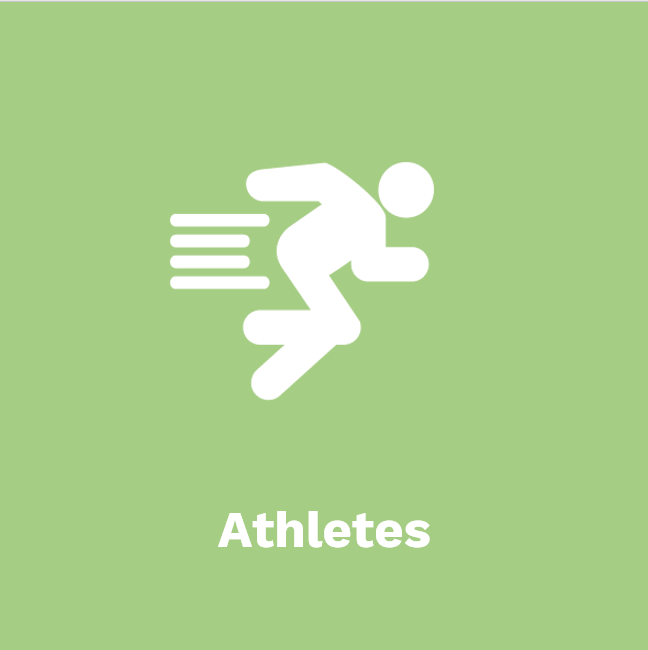

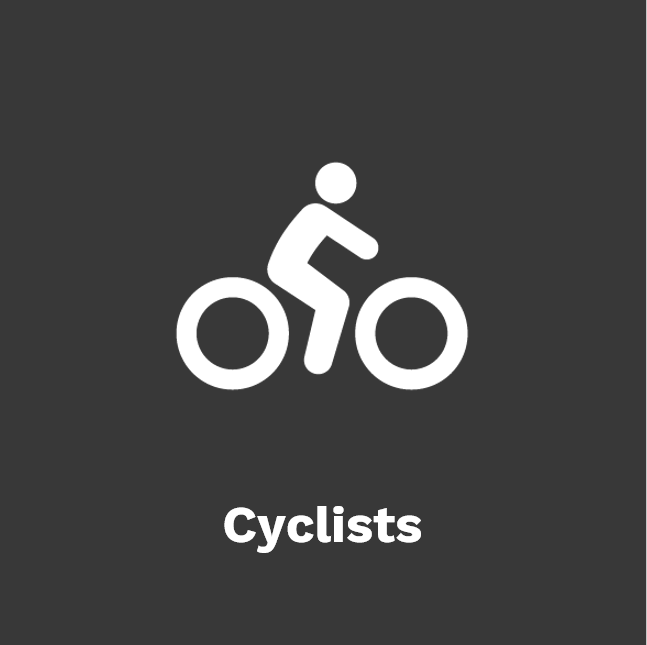

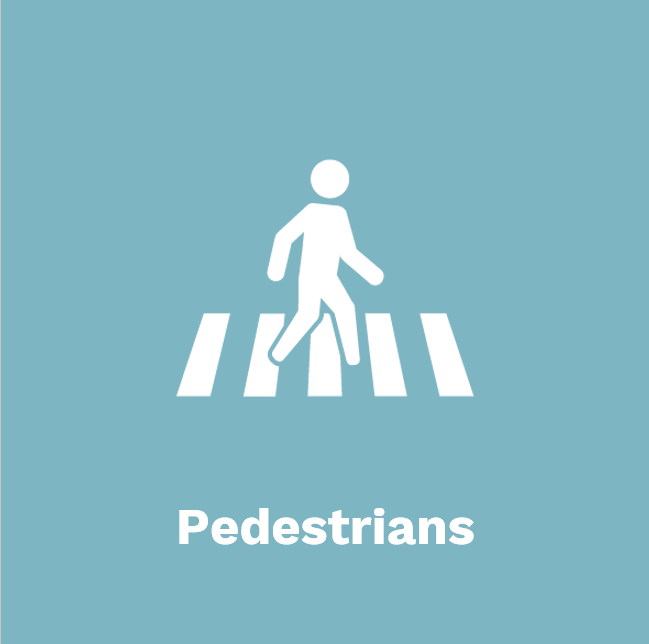

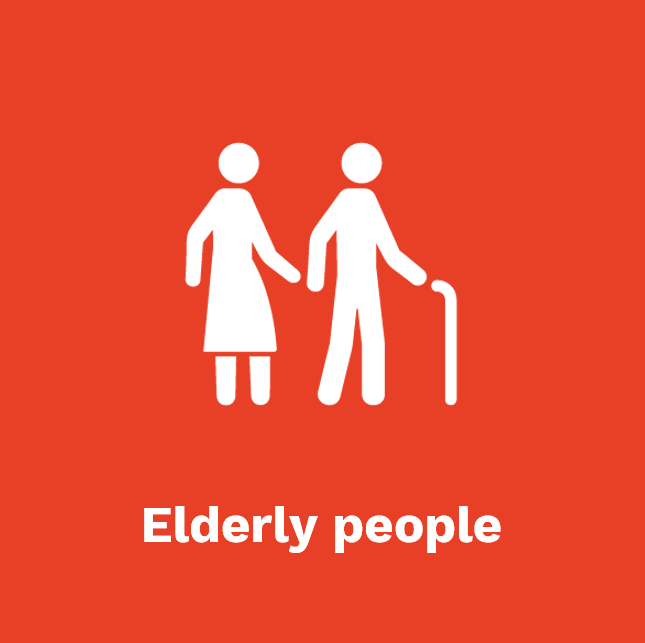

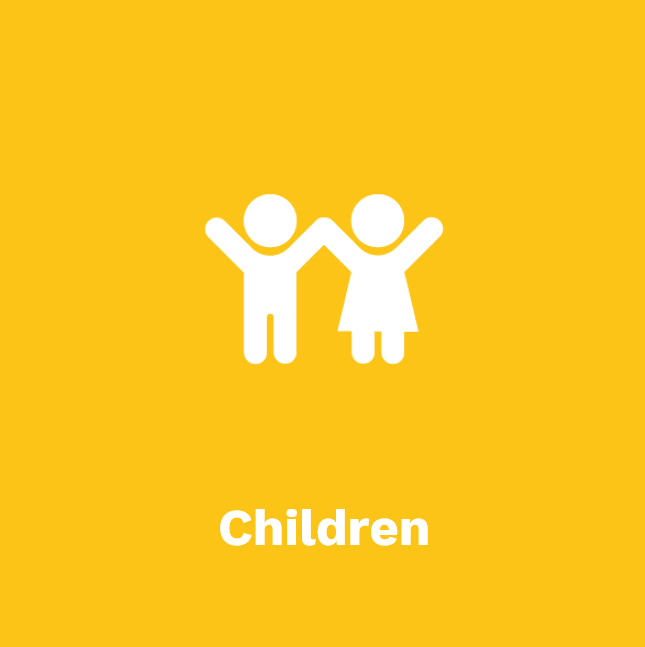

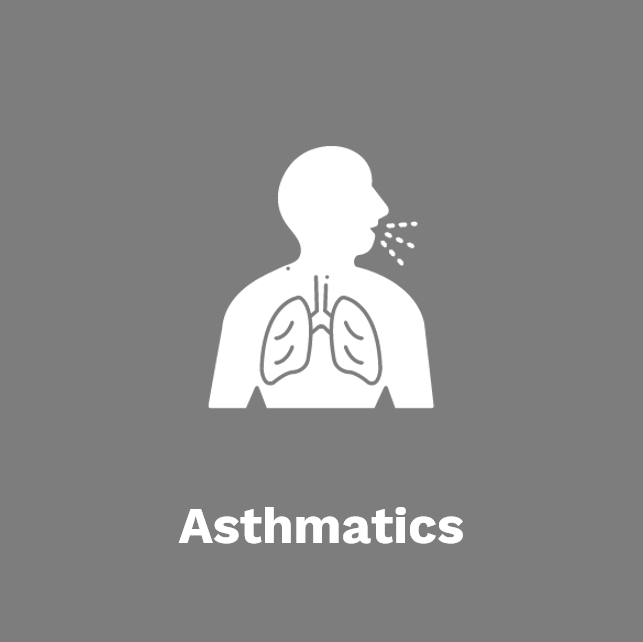


**Tool template 3: Experiment Design canvas**


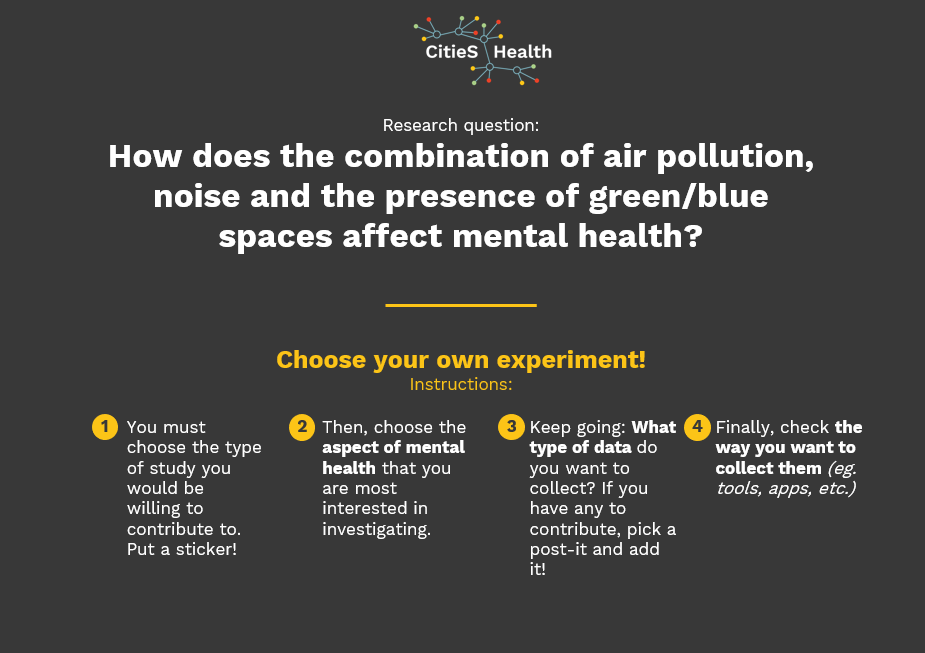


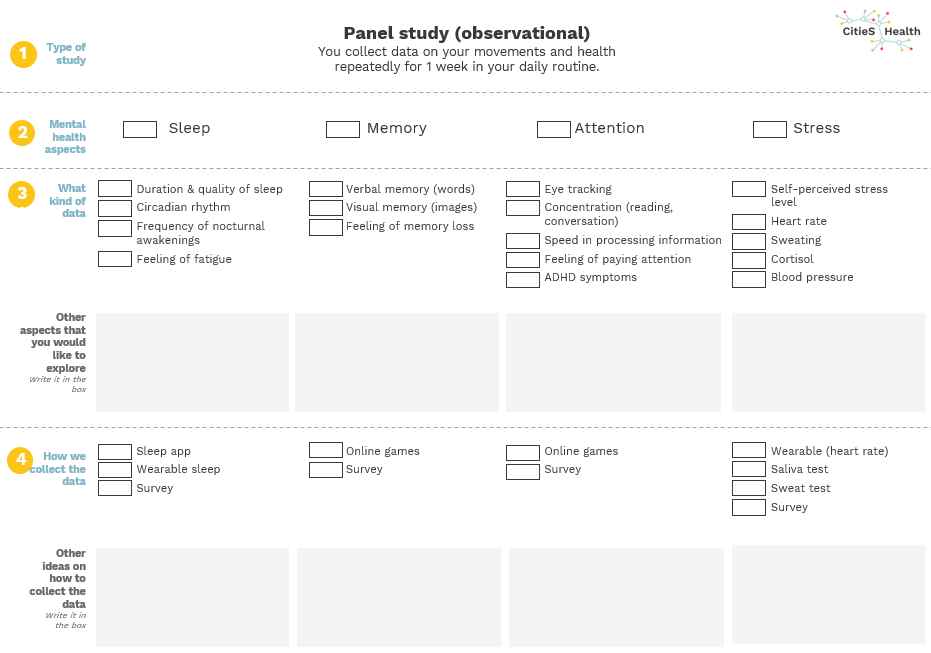


**Table S1.** List of questions from the Barcelona CitieS-Health Pilot Survey 2019.

(English translation from original version)

| Questions | Answers options | |
| --- | --- | --- |
| **Q1:** What age group do you belong to? | - *Less than 18 years old* - *Between 18 to 27 years old* - *Between 28 to 37 years old* | - *Between 38 to 47 years old* - *Between 48 to 64 years old* - *More than 65 years old* |
| **Q2:** In which zone do you live in? (indicate the postal code) | *Technical open-ended question* |  |
| **Q3:** From a scale 0 to 5, how would you rate the quality of air in the community where you live? | *Closed-ended question where respondents select one answer from a scale to 0 to 5 (0= Not polluted, 5=Highly pollluted)* | |
| **Q4:** In which situations do you perceive that air pollution affects you the most? * | - *While walking in the street* - *While doing outdoor sports* - *While going by bicycle* - *While walking with children* | - *While I am at home* - *While I am at work* - *While I am driving* - *While traveling by metro* - *Other* |
| **Q5:** Are you worried about how air pollution affects any of these groups? * | - *Elderly people* - *Children* - *Students* - *People with allergies* - *People with asthma or respiratory problems* | - *Pregnant women* - *Sportsmen/women* - *Deliverymen in motorbike* - *Pedestrians* - *Other* |
| **Q6:** You would like to know how pollution affects … * | - *Hair / Skin* - *Sport performance* - *Respiratory system* - *Digestive system* - *Ageing* - *Heart and arteries* | - *Concentration and development* - *Stress* - *Fertility* - *Mental health* - *Allergies* - *Other* |
| **Q7:** According to your criteria, what are the health effects of air pollution on your health or the others around you? | *Open-ended question* | |
| **Q8:** If you could decide what to investigate concerning air pollution and its health effects, what would you suggest? | *Open-ended question* | |
| **Q9:** Would you like to be part of this project or follow the advances of the investigation? | *Technical open-ended question (respondents’ email)* | |

* Semi-closed ended questions where respondents can select more than one answers from a defined list of choices.

**Table S2.** Translated answers from option ‘Other’ in Q4, Q5 and Q6 for respondents living in the 10 districts of Barcelona.

| **Spanish / Catalan original answers** | **English translation** |
| --- | --- |
| **Question 4** (n = 13) | |
| *Brutìcia mobles* | Dirty furniture |
| *Cuando espero el bus* | When I am waiting for the bus |
| *Cuando hace calor! Me ha llegado a dar asma por salir a vía Laietana en Verano!* | When it is warm! I even had asthma to exit to the street ‘Laietana’ in summer! |
| *Cuando miro Barcelona desde collserola* | When I look at Barcelona from Collserola |
| *En casa, caminando, haciendo deporte, paseando con niños y con mayores* | In my house, when I walk, when I do sport, when I walk with the kids and with elderly people |
| *En las plantas y el minihuerto que cultivo* | In the plants and the little garden |
| *En todas* | In all of them |
| *No percibo la contaminación* | I don’t perceive air pollution |
| *Quan vaig al centre de Barcelona.* | When I go to the center of Barcelona |
| *Quan vaig en moto* | When I go in motorbike |
| *Quan vinc de meu poble a la muntanya* | When I go from my village to the mountain |
| *cuando voy en moto eléctrica* | When I go in electrical motorbike |
| *quan travesso un carrer que acaba de passar un cotxe, bus, camió* | When I cross the street and a car, a bus or a truck have just passed by. |
| **Question 5** (n = 19) | |
| *A Todos* | Everyone |
| *A tots els col·lectius, sobretot els més vulnerables* | To all the collectives, especially the most vulnerable ones |
| *Animales!* | Animals! |
| *El colectivo somos todos* | The collective we are everyone |
| *Per tothom en general* | To everyone in general |
| *Personas y animales sufrimos con la cont* | People and animals |
| *Persones amb malalties cròniques* | People with chronic diseases |
| *Si existe, afecta a todos* | If exists, it affects everyone |
| *Todas las personas* | To everyone |
| *Todo el mundo* | Everyone |
| *Todos* | Everyone |
| *Todos los ciutadadanos* | All citizens |
| *Todos y todas* | Everyone |
| *Todos!* | Everyone |
| *Todos. Humanos y no-humanos* | Everyone. Humans and non-humans |
| *Tothom** | Everyone |
| *Tots* | All |
| **Question 6** (n = 11) | |
| *A totes les anteriors!* | All |
| *Cómo afecta en todas las mencionadas.* | All mentioned before |
| *Cáncer* | Cancer |
| *Esperanza y calidad de vida* | Life expectancy and quality of life |
| *Estado de salud en general* | Health general state |
| *Me preocupa tener que convivir con la contaminación* | It worries me that I have to live with air pollution |
| *Salud mental y al insomnio* | Mental health and insomnia |
| *Salud física y mental* | Physical health and mental health |
| *Todas las opciones* | All the options |
| *Todos* | All |
| *Tot* | All |

*Repeated by three respondents

**Table S3.** List of high frequency health-related and effect-related words (≥ 10 times) from Q7 among respondents living in Barcelona sorted by frequency (English translation from original words).

| **Words** | **n** |
| --- | --- |
| *Respir-* | 312 ^b^ |
| *Card-* | 80 ^b^ |
| *Stress* | 75 ^b^ |
| *Alerg-* | 68 |
| *Development ^a^* | 50 |
| *Quality* | 50 |
| *Skin* | 39 |
| *Ageing* | 37 |
| *Cogni ^a^* | 34 |
| *Cancer* | 32 |
| *Worsen* | 32 ^c^ |
| *Dimin-* | 32 ^c^ |
| *Asma-* | 26 |
| *Premat-* | 21 |
| *Pulmon-* | 20 |
| *Mental* | 19 |
| *Discomfort* | 19 |
| *Fatigue* | 18 |
| *Irritability* | 17 |
| *Capacity* | 17 |
| *Augment-* | 17 ^c^ |
| *Feel* | 16 |
| *Eye* | 16 |
| *Concentration* | 16 |
| *Mortality* | 16 |
| *Head* | 13 |
| *Body* | 13 |
| *Pain* | 11 |
| *Expectancy* | 11 |
| *Exacerbate* | 10 |
| *Circulat-* | 10 |
| *Organism* | 10 |

^a^ “Development” was often expressed as “cognitive development” but not always, thus the two terms were not linked together. However, if linked, the total frequency equals to 84, making this topic in the main health concerns after respiratory health. ^b^ The top three most repeated health-related words. ^c^ The top three most repeated effect-related words.

**Figure S1.** (**a**) Subjective perception of air quality (higher rate indicates a perception of a higher level of air pollution) by district in Barcelona; (**b**) Modeled NO_2_ concentrations in Barcelona in 2017, aggregated at district level. (Ajuntament de Barcelona and Barcelona Regional, 2019).


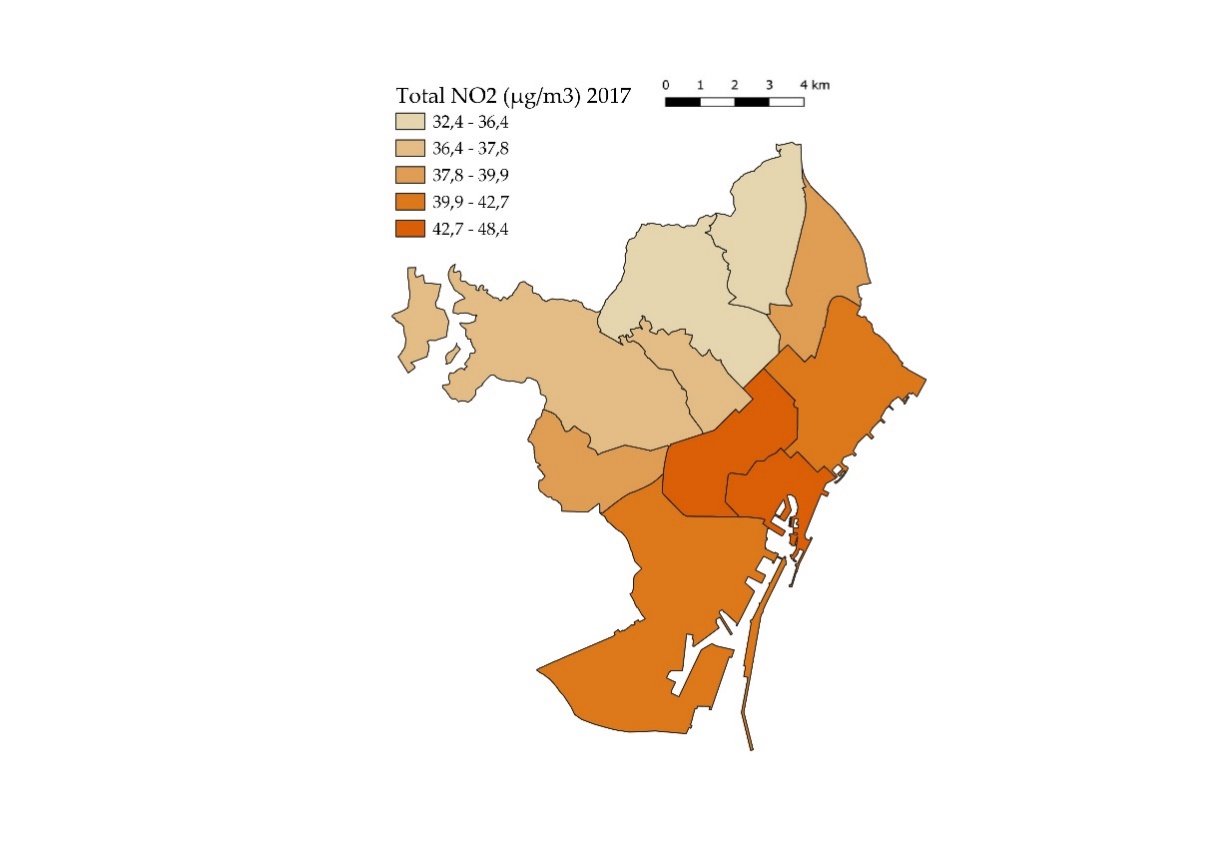

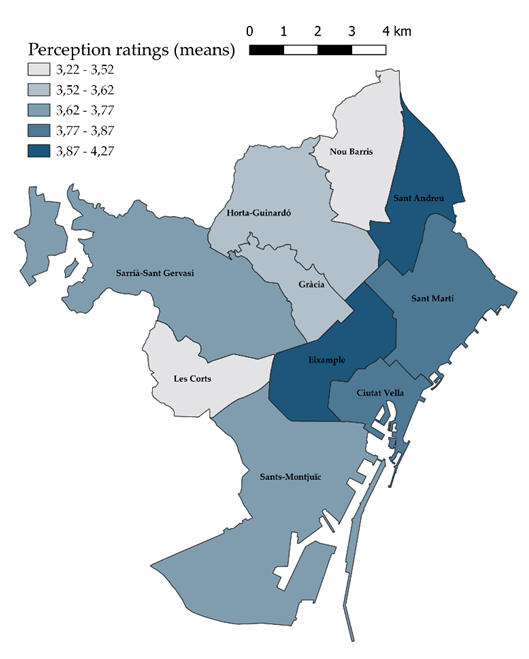


In order of the districts (Eixample, Sant Andreu, Sant Martí, Ciutat Vella, Sants-Montjuïc, Sarrià-Sant Gervasi, Horta-Guinardó, Gràcia, Nou Barris, Les Corts) means of air quality perception are as follow: 3.22, 3.40, 3.55, 3.60, 3.64, 3.73, 3.83, 3.87, 3.88, 4.27.

**Figure S2.** Results^a^ of the co-creation workshop for each type of study design: (a) Panel / Observational, (b) Experimental and (c) Cross-sectional.


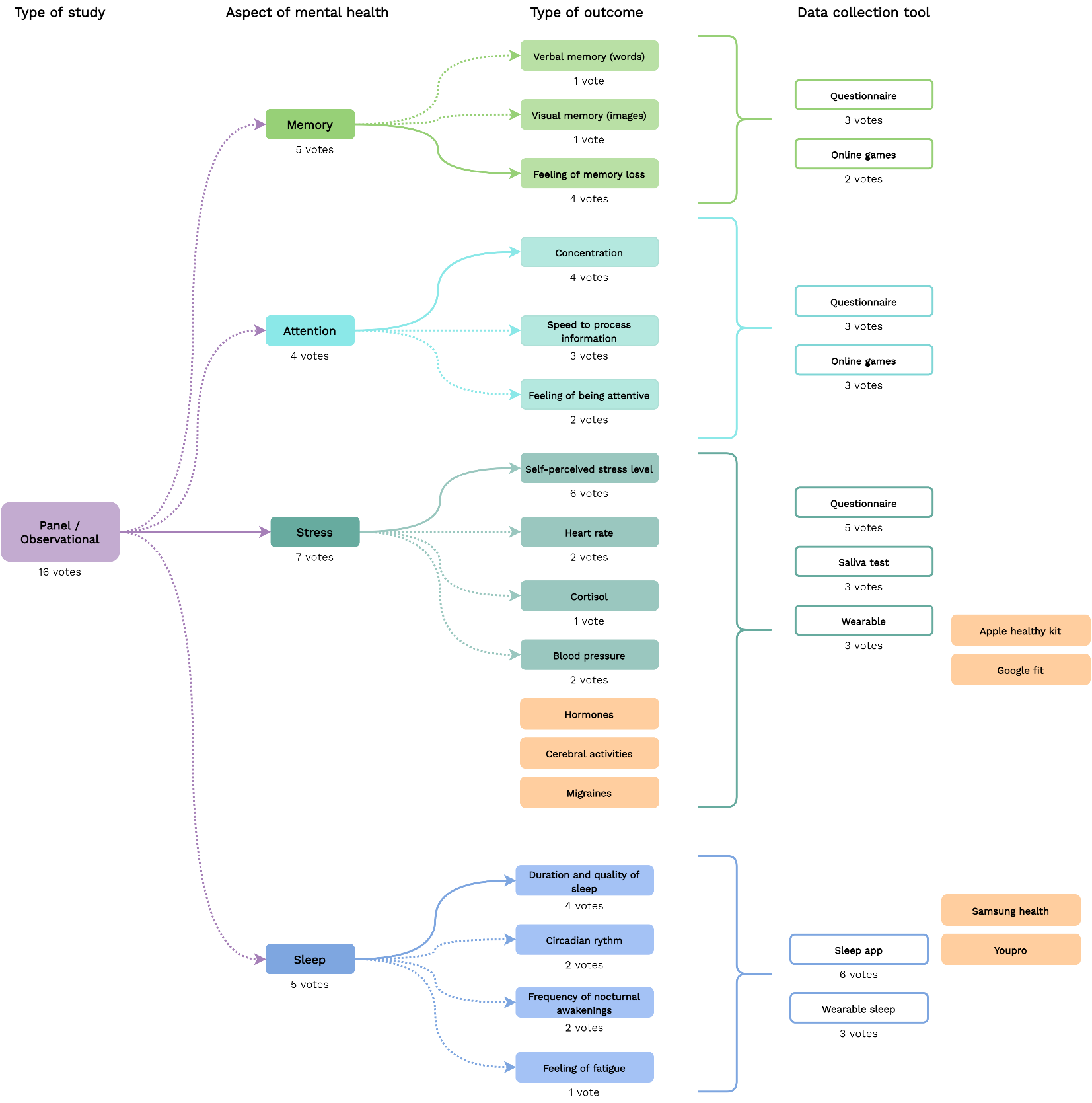


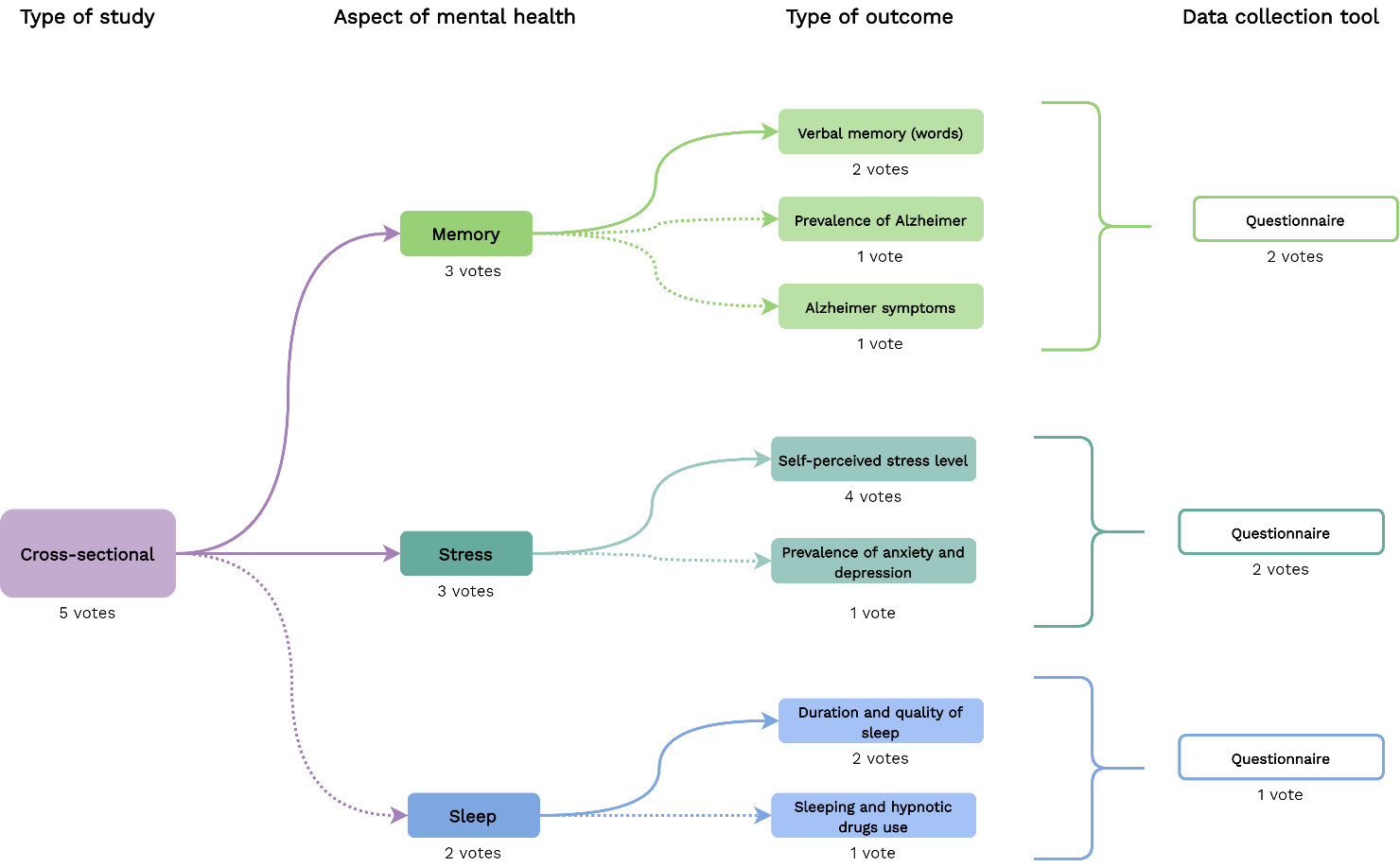

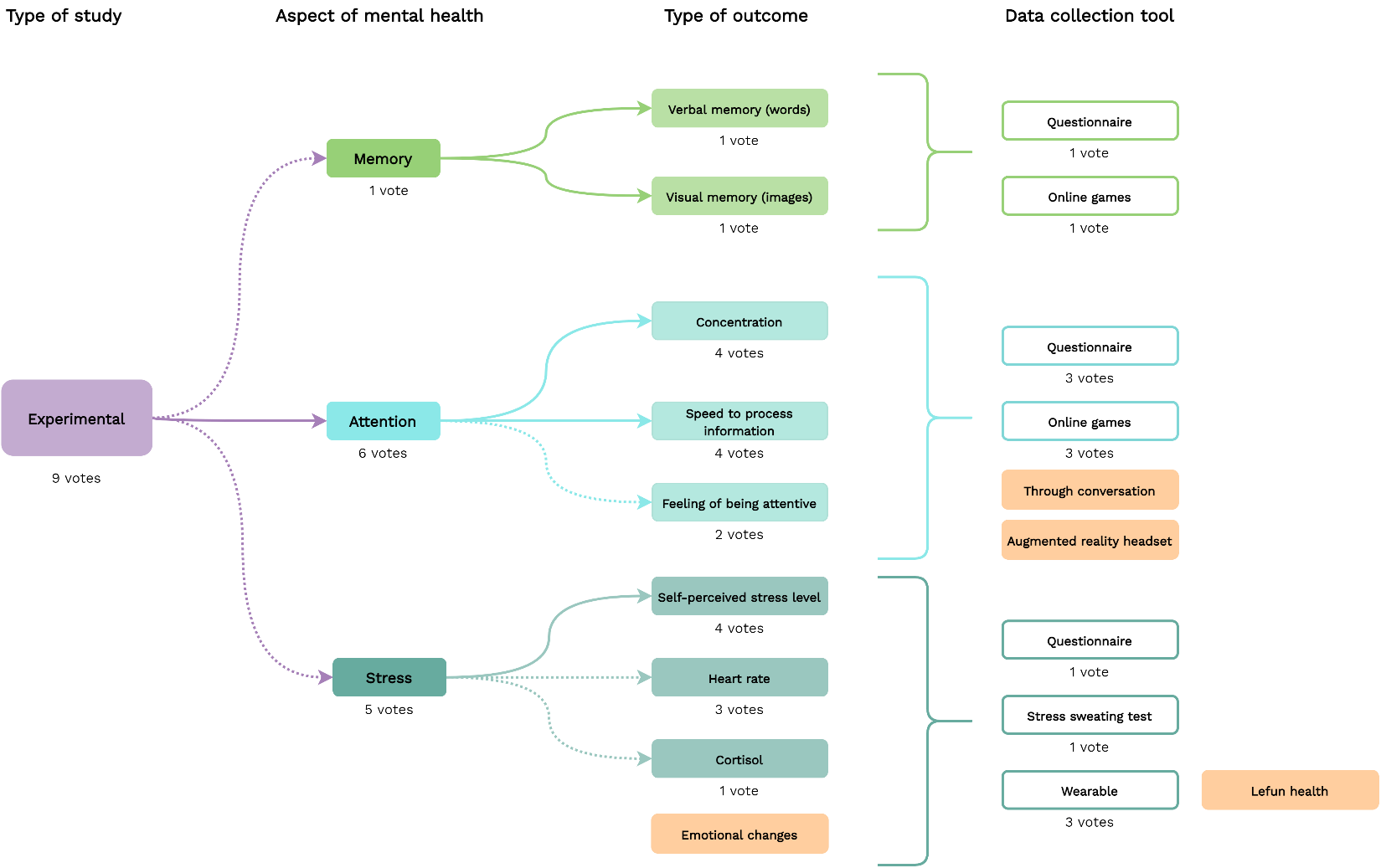


^a^ Results are inconsistent since citizens did not necessarily follow every steps of the activity, and thus lead to several

inconsistencies in the numbers of votes in the Experiment Design Canvas. The most popular options are indicated using the bolded arrows. Suggestions made by the participants are identified using orange blocks.
